# MCTGNet: A Multi-Scale Convolution and Hybrid Attention Network for Robust Motor Imagery EEG Decoding

**DOI:** 10.3390/bioengineering12070775

**Published:** 2025-07-17

**Authors:** Huangtao Zhan, Xinhui Li, Xun Song, Zhao Lv, Ping Li

**Affiliations:** 1 School of Computer Science and Technology, Anhui University, Hefei 230601, China; e23201023@stu.ahu.edu.cn (H.Z.); xinhuili@ahu.edu.cn (X.L.); xun_song@outlook.com (X.S.); kjlz@ahu.edu.cn (Z.L.); 2 The Key Laboratory of Flight Techniques and Flight Safety, Civil Aviation Flight University of China, Deyang 618307, China

**Keywords:** motor imagery, EEG decoding, cross-session generalization, Kolmogorov–Arnold Network

## Abstract

Motor imagery (MI) EEG decoding is a key application in brain–computer interface (BCI) research. In cross-session scenarios, the generalization and robustness of decoding models are particularly challenging due to the complex nonlinear dynamics of MI-EEG signals in both temporal and frequency domains, as well as distributional shifts across different recording sessions. While multi-scale feature extraction is a promising approach for generalized and robust MI decoding, conventional classifiers (e.g., multilayer perceptrons) struggle to perform accurate classification when confronted with high-order, nonstationary feature distributions, which have become a major bottleneck for improving decoding performance. To address this issue, we propose an end-to-end decoding framework, MCTGNet, whose core idea is to formulate the classification process as a high-order function approximation task that jointly models both task labels and feature structures. By introducing a group rational Kolmogorov–Arnold Network (GR-KAN), the system enhances generalization and robustness under cross-session conditions. Experiments on the BCI Competition IV 2a and 2b datasets demonstrate that MCTGNet achieves average classification accuracies of 88.93% and 91.42%, respectively, outperforming state-of-the-art methods by 3.32% and 1.83%.

## 1. Introduction

Brain–computer interfaces (BCIs) are attracting increasing interest for their potential to enable direct communication between the human brain and external devices [1,2,3]. Among various paradigms, motor imagery (MI) has emerged as a particularly appealing modality [4,5]. By mentally rehearsing limb movements without any physical execution, users activate sensorimotor brain regions and generate EEG patterns that can be decoded by BCI systems. Compared with other BCI paradigms, MI offers a more intuitive and self-paced interaction mechanism, making it well-suited for long-term neurorehabilitation, intelligent control, and assistive communication systems.

Despite growing interest in MI-based BCI systems, decoding MI-EEG signals remains a challenging task due to their intrinsic variability and complex temporal, spectral, and spatial dynamics. In particular, MI-EEG signals exhibit pronounced non-stationarity across sessions, often caused by changes in user state such as fluctuations in attention, mental fatigue, or emotional arousal [6,7]. Such variability leads to substantial distribution shifts, which severely undermine the generalization and robustness of decoding models in cross-session scenarios. To this end, numerous approaches have been proposed, ranging from feature extraction techniques to advanced model architectures and novel MI paradigms.

Among these efforts, multi-scale feature modeling [8,9,10] has been widely explored as a promising strategy for enhancing MI decoding performance. By capturing EEG rhythms at different temporal and spectral resolutions, this approach enables the extraction of diverse neural signatures associated with motor imagery, offering a more robust and informative representation of the underlying brain activity. However, despite their effectiveness, existing methods often struggle to fully exploit multi-scale features. A primary limitation lies in their reliance on conventional classifiers, such as multilayer perceptrons (MLPs), which lack the expressive capacity required to model the high-order, nonstationary nature of MI-EEG feature distributions [6,7]. Moreover, heterogeneous information structures, such as local and global features, also display complex, dynamic patterns, further complicating the classification process.

To address the aforementioned modeling bottlenecks, this paper proposes an end-to-end decoding framework called **MCTGNet**, whose core idea is to formulate the classification process as a high-order function approximation task, thereby structurally overcoming the expressive limitations of traditional classifiers. Unlike conventional classifiers that rely on stacked linear-affine transformations and nonlinear activation functions, we introduce the Kolmogorov–Arnold Transformer (KAT) [11], which decomposes multivariate mappings into compositions of univariate functions. This formulation enhances the model’s ability to represent complex nonlinear structures. As a result, the proposed method not only improves the discriminative capacity for task labels, but also strengthens the unified modeling of heterogeneous feature structures such as local and global representations, thereby significantly improving the quality of multi-scale feature fusion and the generalization performance under cross-session conditions.

However, to implement MCTGNet into a real system, some challenges must be faced:

- **Challenge 1: Limited perceptual range of feature extractors.** MI-EEG signals contain multiple neural rhythms across various frequency bands, each reflecting distinct stages of motor imagination. However, existing models often rely on fixed receptive fields or single-scale operations, limiting their ability to fully capture the diverse temporal structures embedded across frequency components. This constraint reduces the comprehensiveness of the extracted feature representations and compromises decoding performance.
- **Challenge 2: Inconsistent representation of heterogeneous features.** MI-EEG signals exhibit both local transient dynamics and global cross-channel coordination, which differ in spatial scale, semantic granularity, and temporal patterning. Conventional classifiers lack mechanisms to unify these heterogeneous patterns in a consistent representational space, thereby hindering the construction of a shared discriminative function and weakening the system’s robustness.
- **Challenge 3: Insufficient modeling capacity for discriminative functions.** Due to cross-session variability, MI-EEG features often manifest as highly nonlinear, nonstationary, and distributionally divergent. Traditional classifiers such as MLPs struggle to capture the complex high-order mappings required to distinguish task labels under such conditions. This limits the model’s ability to generalize across sessions and maintain stable performance.

To address the aforementioned challenges, MCTGNet introduces tailored designs at both the feature extraction and classification stages. For Challenge 1, a multi-scale perception module is developed to construct a broad spectral representation space by applying parallel convolutions with varying receptive fields, thereby capturing diverse EEG rhythms. For Challenge 2 and Challenge 3, a group-rational Kolmogorov–Arnold Network (GR-KAN) is employed, which models the classification process as a high-order function approximation task. Specifically, GR-KAN distinguishes heterogeneous structures such as local and global features via grouped mapping mechanisms, improving discrimination under complex spatial hierarchies (Challenge 2). Moreover, its high-order expressive structure enables the modeling of nonlinear decision boundaries, enhancing the separability of MI task representations and improving robustness under cross-session conditions (Challenge 3).

The major contributions of this work are summarized as follows:

- We introduce a novel Kolmogorov–Arnold Network (KAN) formulation to motor imagery EEG decoding, by casting the classification task as a high-order function approximation problem. This perspective enhances the model’s ability to capture complex, nonlinear, and nonstationary distributions in EEG feature space, overcoming the expressive limitations of conventional MLP-based classifiers.
- We propose an end-to-end neural architecture called MCTGNet, which integrates a Multi-Scale Perception Module (MPE-CNN) for extracting neural rhythms across diverse temporal scales, and a group-rational KAN (GR-KAN) for structured, high-capacity decision modeling. This design provides a principled solution to the challenge of heterogeneous feature representation and nonlinear separability in cross-session MI decoding.
- We further incorporate a Hybrid Local-Global Attention mechanism (LG-KAT) to enhance temporal feature integration by explicitly modeling both fine-grained local fluctuations and global contextual patterns in EEG sequences. This enables more robust and semantically consistent fusion across temporal hierarchies.

We emphasize the novel contribution of MCTGNet in providing a structured solution to the challenges of multi-scale feature fusion, heterogeneous feature representation, and high-order classification in MI-EEG decoding, with the potential to become a new baseline for MI-EEG decoding. The source code has been released at https://github.com/huangt126/MCTGNet, accessed on 23 June 2025.

The remainder of this paper is organized as follows: Section 2 reviews the related work; Section 3 introduces the preliminary knowledge; Section 4 presents the detailed architecture and training strategy of MCTGNet; Section 5 reports the experimental results and performance comparison. We discuss some limitations of this paper in Section 6 and conclude this paper in Section 7.

## 2. Related Work

### 2.1. Feature Extraction in MI-EEG Decoding

Effective feature extraction plays a crucial role in MI-EEG decoding, as it directly influences the system’s ability to identify discriminative neural patterns. Early methods primarily relied on traditional signal processing and classical machine learning techniques [12]. Among them, Common Spatial Pattern (CSP) and its extensions [13,14,15,16] have been widely used to extract frequency-specific features by maximizing inter-class variance. Meanwhile, time-frequency analysis approaches, such as the continuous wavelet transform (CWT) [17], have been applied to model the nonstationary nature of EEG signals. However, these approaches depend heavily on handcrafted features and domain expertise, limiting their adaptability to complex neural dynamics.

With the rapid advancement of deep learning, convolutional neural networks (CNNs) have achieved notable success in learning spatiotemporal representations directly from raw EEG signals [18,19]. Nevertheless, most CNN-based models utilize fixed-scale convolutional kernels, which constrain their capacity to fully capture the multi-frequency characteristics intrinsic to motor imagery tasks. To address this limitation, multi-scale feature modeling has emerged as a promising strategy. By incorporating parallel convolutional branches with varied receptive fields, these models can extract neural patterns across different temporal and frequency scales. Such architectures have demonstrated improved robustness and classification performance, particularly in cross-session decoding scenarios [20].

### 2.2. Feature Fusion Strategies

Building upon effective feature extraction, the integration of multi-dimensional EEG features plays a pivotal role in enhancing motor imagery (MI) decoding performance. Early methods typically employed handcrafted fusion techniques—such as discrete wavelet transforms and filter banks—to combine frequency-specific features across spatial and temporal dimensions [21,22]. While conceptually straightforward, these approaches lacked adaptability and struggled with generalization due to the nonstationary and subject-specific nature of EEG signals.

Recently, the transformer model has shown excellent performance in natural language processing and computer vision for its long-range dependency modeling and efficient parallel computing [23].Transformer and its variants have been applied across various fields with significant success. For instance, Roy et al. [24] proposed ’SimPoolFormer’, which replaces multi-headed self-attention with SimPool and integrates linear attention for hyperspectral image classification. Kasoju et al. [25] optimized Transformer models for low-latency inference, achieving efficiency gains without sacrificing accuracy. Shaker et al. [26] introduced SwiftFormer, a Transformer variant with an efficient additive attention mechanism tailored for real-time mobile vision applications, optimized for resource-constrained devices.

Similarly, transformer-based models have been gaining popularity in EEG decoding [27,28,29,30]. These models are particularly advantageous as they benefit from an extended receptive field, enabling them to capture long-range dependencies in MI-EEG signals. Certainly, Transformer-based fusion architectures have been proposed to learn complex interactions across spatial, temporal, and spectral domains [31,32,33,34]. For instance, Zhou et al. [35] introduced an attention-driven hybrid network that dynamically emphasizes salient spatial and temporal features, enhancing discriminative representation learning. The Adaptive Dual-Feature Fusion Convolutional Neural Network (ADFCNN) [20] jointly captures spectral and spatial patterns via dual-scale convolution and attention mechanisms, achieving robust performance under nonstationary conditions. Similarly, Multi-Scale Attention Fusion Network (MAFNet) [32] integrates multi-resolution features through adaptive weighting guided by attention, facilitating more flexible representation across diverse frequency bands.

Multi-scale fusion has thus emerged as a promising direction. By employing parallel branches with varied kernel sizes or receptive fields, such architectures aim to capture neural patterns across fine and coarse temporal and frequency resolutions [36,37]. However, despite these advancements, current fusion frameworks still face structural challenges in aligning heterogeneous representations—particularly in distinguishing and integrating local and global features. This semantic mismatch limits the consistency of the fused feature space and constrains the full potential of multi-scale learning.

### 2.3. Classifier for MI-EEG Decoding

In MI-EEG decoding, the classifier serves as the final and critical component, responsible for mapping extracted neural representations to task-specific labels. With the advancement of deep learning, convolutional neural networks (CNNs) have been extensively employed in motor imagery classification tasks due to their effectiveness in capturing spatial patterns. Representative architectures such as DeepConvNet and EEGNet have demonstrated competitive performance across various benchmarks [18,19]. In parallel, recurrent neural networks (RNNs) and their variants—such as LSTM and GRU—have been utilized for modeling temporal dependencies, although their limited training efficiency and poor scalability have restricted broader applicability [38,39].

Despite the progress in feature extraction and fusion, most decoding frameworks still adopt conventional multilayer perceptrons (MLPs) for classification. MLPs perform input-to-output mapping through stacked linear transformations and nonlinear activations, and are theoretically capable of approximating any continuous function [40]. However, in practice, traditional MLPs often fall short when confronted with the nonlinear, nonstationary, and structurally heterogeneous distributions typical of MI-EEG data. Moreover, the dense connectivity in MLPs results in significant parameter overhead, increasing the risk of overfitting and limiting generalization.

Recently, Kolmogorov–Arnold Networks (KANs) have emerged as a promising alternative to traditional multilayer perceptrons (MLPs) in various domains. For example, Han et al. [41] applied KANs to hyperspectral image classification. Similarly, Galitsky et al. [42] optimized KANs for word-level explainable meaning representation, leveraging their interpretability to improve model transparency compared to MLPs. Furthermore, Hu et al. [43] introduced the Kolmogorov–Arnold Classifier (KAC) for continual learning, replacing MLP structures with KANs to alleviate catastrophic forgetting in real-time learning scenarios. Despite these advancements, KANs still face a significant challenge: their relatively high computational cost. This limits their practical application, especially in resource-constrained environments. In the context of MI-EEG decoding, the computational demands of KANs present particular difficulties for real-time classification systems. These systems require low-latency processing to provide prompt feedback, but the high computational overhead of KANs can lead to delays, impeding their deployment in real-time applications.

## 3. Preliminary

### 3.1. Transformer Encoder

**Figure 1:**
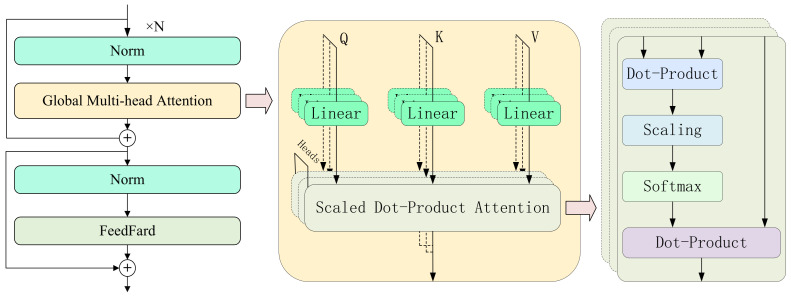
The structure of Transformer encoder.

The Transformer is a powerful model for sequence processing that utilizes the self-attention mechanism to capture global dependencies [23]. In MI-EEG classification, the Transformer encoder is commonly employed to perform deep feature extraction on the processed features from earlier network layers. Its typical structure is illustrated in Figure 1.

For an input sequence $R^{n\times d}$, where *n* represents the number of time steps and *d* the feature dimension, the Transformer generates Query, Key, and Value triplets by performing linear projections:

(1) $$Q=XW_{Q},\;K=XW_{K},\;V=XW_{V}\;\left(W_{Q},W_{K},W_{V}\in R^{d\times d_{k}}\right)$$

The attention matrix $A\in R^{n\times n}$ is then computed using the scaled dot-product attention mechanism, which quantifies the correlation strength between any two time steps:

(2) $$A=\mathrm{softmax}\left(\frac{QK^{⊤}}{\sqrt{d_{k}}}\right),\;\mathrm{Output}=AV$$

The scaling factor $\sqrt{d_{k}}$ ensures numerical stability during the calculation of dot products with high-dimensional vectors. To enhance feature diversity, the Multi-Head Attention (MHA) mechanism splits the input into *h* independent subspaces, computes attention for each subspace in parallel, and concatenates the resulting projections:

(3) $${\mathrm{head}}_{i}=\mathrm{Attention}\left(XW_{Q}^{i},XW_{K}^{i},XW_{V}^{i}\right),\;\mathrm{MHA}\left(X\right)=\left[{\mathrm{head}}_{1};{\mathrm{head}}_{2};\ldots ;{\mathrm{head}}_{h}\right]$$

To incorporate positional information into the sequence, sinusoidal positional encoding is applied to provide absolute temporal context:

(4) $${\mathrm{PE}}_{\left(\mathrm{pos},2i\right)}=\sin\left(\frac{\mathrm{pos}}{10000^{2i/d}}\right),\;{\mathrm{PE}}_{\left(\mathrm{pos},2i+1\right)}=\cos\left(\frac{\mathrm{pos}}{10000^{2i/d}}\right)$$

The encoder then applies residual connections followed by layer normalization (LayerNorm), and uses a feed-forward network (FFN) to perform a nonlinear transformation of the features:

(5) $$\mathrm{FFN}\left(x\right)=\mathrm{GELU}\left(xW_{1}+b_{1}\right)W_{2}+b_{2}$$

### 3.2. Kolmogorov–Arnold Representation Theorem and Network

The Kolmogorov–Arnold Representation Theorem asserts that any continuous multivariate function defined on a bounded domain can be expressed as a combination of a finite number of univariate continuous functions and addition [44]. Specifically, for a smooth function $f:{\left[0,1\right]}^{n}\to R$, its representation is given by:

(6) $$f\left(x_{1},\ldots ,x_{n}\right)=\sum_{q=1}^{2n+1}\Phi_{q}\left(\sum_{p=1}^{n}\phi_{q,p}\left(x_{p}\right)\right)$$

where $\phi_{q,p}:\left[0,1\right]\to R$ are continuous univariate functions for the input dimensions, and $\Phi_{q}:R\to R$ are continuous univariate functions for the output dimension.

This theorem can also be expressed in matrix form as follows:

(7) $$f\left(x\right)=\Phi_{\mathrm{out}}\circ \Phi_{\mathrm{in}}\circ x,$$

where the input transformation matrix $\Phi_{\mathrm{in}}$ and the output transformation matrix $\Phi_{\mathrm{out}}$ are defined as:

(8) $$\Phi_{\mathrm{in}}=\left[\begin{array}{ccc} \phi_{1,1}\left(\cdot \right) & \ldots & \phi_{1,n}\left(\cdot \right)\\ \vdots & \ddots & \vdots \\ \phi_{2d+1,1}\left(\cdot \right) & \ldots & \phi_{2d+1,d}\left(\cdot \right) \end{array}\right],\;\Phi_{\mathrm{out}}=\left[\begin{array}{ccc} \Phi_{1}\left(\cdot \right) & \ldots & \Phi_{2d+1}\left(\cdot \right) \end{array}\right]$$

Building upon this theorem, the Kolmogorov–Arnold Network (KAN) was proposed [11]. The core idea of KAN is to replace the fixed activation functions used in traditional neural networks with learnable edge activation functions. For a KAN layer with $d_{\mathrm{in}}$-dimensional input and $d_{\mathrm{out}}$-dimensional output, its computational form is:

(9) $$f\left(x\right)=\Phi \circ x=\left[\begin{array}{ccc} \sum_{i=1}^{d_{\mathrm{in}}}\phi_{i,1}\left(x_{i}\right) & \ldots & \sum_{i=1}^{d_{\mathrm{in}}}\phi_{i,d_{\mathrm{out}}}\left(x_{i}\right) \end{array}\right]$$

where the parameterized matrix Φ contains all the edge activation functions:

(10) $$\Phi =\left[\begin{array}{ccc} \phi_{1,1}\left(\cdot \right) & \ldots & \phi_{1,d_{\mathrm{in}}}\left(\cdot \right)\\ \vdots & \ddots & \vdots \\ \phi_{d_{\mathrm{out}},1}\left(\cdot \right) & \ldots & \phi_{d_{\mathrm{out}},d_{\mathrm{in}}}\left(\cdot \right) \end{array}\right]$$

In practice, each activation function ϕ(x) is a linear combination of the SiLU function and B-spline functions:

(11) $$\phi \left(x\right)=w_{b}\cdot \mathrm{SiLU}\left(x\right)+w_{s}\cdot \mathrm{spline}\left(x\right)$$

where $\mathrm{SiLU}\left(x\right)=\frac{x}{1+e^{-x}}$ is the self-gated activation function, and $\mathrm{spline}\left(x\right)=\sum_{i}c_{i}B_{i}\left(x\right)$ represents the B-spline basis function expansion.

The entire KAN network is constructed by stacking multiple layers of composite transformations:

(12) $$\mathrm{KAN}\left(x_{0}\right)=\Phi_{L-1}\circ \Phi_{L-2}\circ \ldots \circ \Phi_{0}\circ x_{0}$$

## 4. Methodology

In this section, we introduce our proposed end-to-end MI-EEG decoding model, **MCTGNet**. Section 4.1 provides an overview of the overall architecture, followed by detailed descriptions of its main modules.

### 4.1. Algorithm Overview

**Figure 2:**
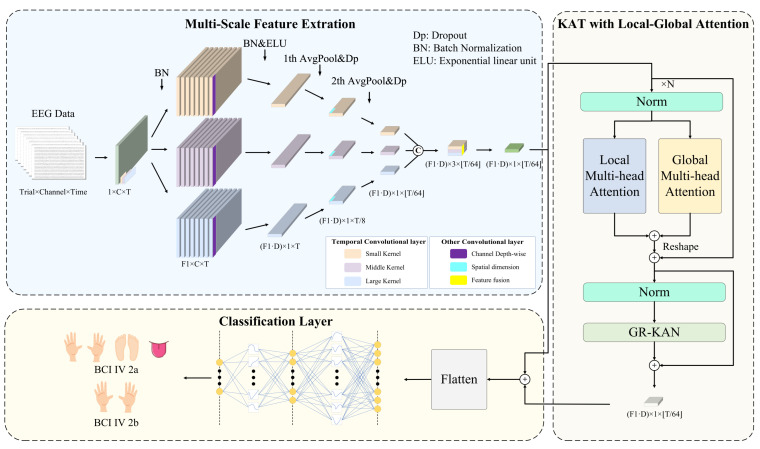
The framework consists of three core modules: the multi-scale patch embedding convolutional module, the local-global attention mechanism encoder based on KAT, and the classification layer based on GR-KAN.

Figure 2 illustrates the architecture of MCTGNet. The model comprises three functional modules. The Multi-Scale Feature Extraction Module (MPE-CNN) captures temporal and spectral patterns at different scales by applying parallel convolutions with varying kernel sizes, enabling rich representation of neural rhythms. The KAT with Local-Global Attention Module (LG-KAT) integrates local temporal details and global contextual cues through a hybrid attention mechanism, enhancing the model’s ability to distinguish fine-grained neural patterns. Finally, the Classification Layer Module employs a high-capacity decision function to map the fused features into task labels, supporting robust decoding across complex conditions. In the following subsections, we present a detailed description of each module.

### 4.2. Multi-Scale Feature Extraction Module

MI-EEG signals exhibit diverse neural activity patterns across different frequency bands. This is particularly evident in MI tasks, where neural rhythms display strong nonstationarity and multi-scale characteristics. Traditional single-scale convolutional structures often struggle to fully extract key features from different frequency ranges, thereby limiting decoding performance. To address this issue, we propose a multi-scale feature extraction module that adopts a multi-branch convolutional design. It consists of three parallel temporal processing paths tailored to model low-frequency (long time window), medium-frequency (moderate window), and high-frequency (short window) neural dynamics. The goal is to construct a cross-frequency, cross-scale neural representation space, enhancing the model’s ability to capture complex EEG patterns and providing more discriminative inputs for the subsequent attention and classification modules.

Specifically, the raw EEG signal is passed through three parallel temporal convolution branches, each with a distinct kernel size $K_{1}=\left\{K_{S},K_{M},K_{L}\right\}$ to capture neural oscillations at multiple temporal scales. Smaller kernels are effective in detecting high-frequency, short-duration patterns, while larger kernels capture long-range, low-frequency dynamics. Each branch outputs a set of frequency-domain feature maps using $F_{1}$ convolutional filters, encoding neural information specific to its receptive scale.

Next, spatial patterns across EEG channels are modeled using depthwise separable convolutions with kernel size (C,1), where *C* denotes the number of input channels. The output dimensionality is expanded via a depth multiplier *D*, allowing richer spatial representations to emerge. To reduce temporal redundancy and computational burden, two stages of average pooling with stride parameters $P_{1}$ and $P_{2}$ are sequentially applied along the temporal axis. These operations compress the temporal resolution while preserving salient features, preparing the signal for downstream processing.

To further strengthen short- and medium-range temporal modeling, a convolutional layer with kernel size $K_{2}$ is inserted after the first pooling operation. This step enables the network to better capture semantic dependencies within a typical window of approximately 500 ms, enhancing sensitivity to transient MI-related patterns.

Finally, the outputs of all three convolution branches are concatenated along the channel dimension, forming a joint feature representation that integrates temporal characteristics across multiple scales. A 1×1 convolution is then applied for compact fusion and channel compression, effectively learning cross-scale interaction weights and reducing redundancy. The fused feature maps are reshaped into a sequential format (L,E), where *L* is the temporal length and *E* is the embedding dimension, serving as the input to the subsequent module. The key hyperparameter settings for this module are summarized in Table 1.

### 4.3. KAT with Local-Global Attention Module

After the multi-scale feature extraction module constructs rich frequency-domain and temporal features, challenges still remain in modeling cross-scale temporal dependencies and preserving local details. These difficulties primarily stem from the inherent limitations of traditional Transformer architectures, where the commonly used MLPs exhibit insufficient expressive power and weak nonlinearity. To further enhance the model’s ability to capture complex motor imagery EEG patterns, we propose a KAT with Local-Global Attention module. The core idea of this module is to introduce a dual-branch attention mechanism that explicitly models both fine-grained short-term dynamics and long-range global dependencies, while leveraging the KAT architecture to improve the model’s nonlinear representation capacity and overall expressiveness.

As illustrated in Figure 2, the module is composed of three key substructures. First, the local attention branch extracts multi-scale temporal dynamics using 1D convolutions with different kernel sizes and incorporates them into the attention computation, thereby enhancing sensitivity to short-term nonstationary rhythms. Second, the global attention branch directly applies standard self-attention to capture long-range temporal dependencies from the input sequence. Finally, GR-KAN layers replace the traditional MLPs, utilizing grouped rational activation functions to achieve improved nonlinearity and parameter efficiency. This design enhances the model’s capacity for capturing intricate EEG dynamics while maintaining computational efficiency.

**Figure 3:**
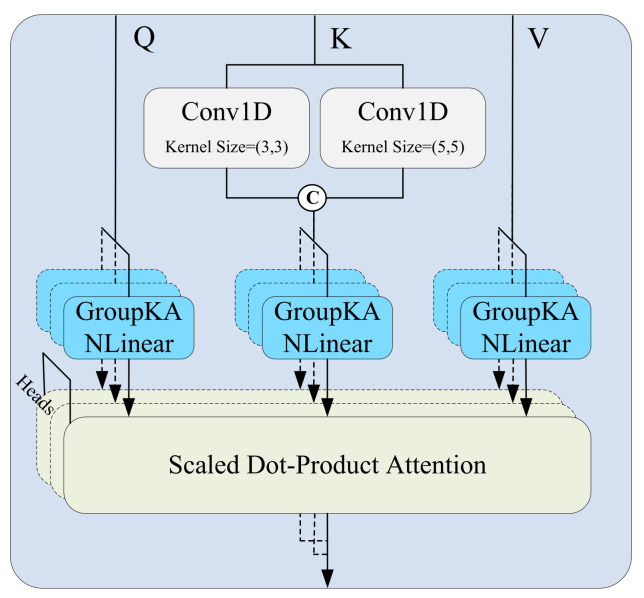
The structure of local attention.

Specifically, in the local attention path, to effectively capture dynamic patterns at multiple time scales, the input feature map $X\in R^{B\times C\times L}$ (where *B* is the batch size, *C* the number of channels, and *L* the temporal length) is processed through several parallel 1D convolution branches, each using a different kernel size $k_{i}$. Zero-padding is applied to preserve the temporal dimension (as shown in Figure 3). Each convolution operation is defined as:

(13) $$y_{i}=\mathrm{Conv}1D\left(X,k_{i},p_{i}\right)$$

where $k_{i}$ denotes the kernel size and $p_{i}$ the corresponding padding. The resulting feature maps $y_{i}$ are concatenated along the channel dimension to construct a multi-scale representation for the key matrix:

(14) $${\mathrm{Key}}_{\mathrm{local}}=\mathrm{Concat}\left(y_{1},y_{2}\right)$$

These representations are combined with the query and value matrices and passed into the standard scaled dot-product attention mechanism to produce local attention outputs:

(15) $${\mathrm{Attention}}_{\mathrm{local}}=\mathrm{Softmax}\left(\frac{QK_{\mathrm{local}}^{⊤}}{\sqrt{d_{k}}}\right)\cdot V$$

Meanwhile, the global attention path uses the same input features to directly generate *Q*, $K_{\mathrm{global}}$, and *V* via linear projections, and computes attention as:

(16) $${\mathrm{Attention}}_{\mathrm{global}}=\mathrm{Softmax}\left(\frac{QK_{\mathrm{global}}^{⊤}}{\sqrt{d_{k}}}\right)\cdot V$$

The local and global attention outputs are fused via element-wise addition:

(17) $$X_{\mathrm{att}}={\mathrm{Attention}}_{\mathrm{local}}+{\mathrm{Attention}}_{\mathrm{global}}$$

On top of this fused representation, we introduce the GR-KAN module to replace conventional MLPs for nonlinear transformation. GR-KAN reformulates classification as a high-order function approximation using group-wise rational functions, which replace the B-spline bases in traditional KANs to enhance expressiveness and support efficient parallel computation on modern GPUs. To reduce parameter overhead, it shares activation weights and basis functions within neuron groups, while variance-preserving initialization ensures stable activation flow across layers. The learnable activation function is defined as the safe Padé unit:

(18) $$\phi \left(x\right)=w\cdot \frac{P(x)}{1+|Q(x)|}$$

where $P\left(x\right)=\sum a_{i}x^{i}$ and $Q\left(x\right)=\sum b_{i}x^{i}$ are learnable polynomials, and *w* is a scaling coefficient. This formulation avoids division-by-zero and enhances the expressive power of the network.

To reduce parameter size and computational cost, GR-KAN adopts a grouping mechanism. The input vector is divided into *g* groups, each with $d_{g}=d_{\mathrm{in}}/g$ dimensions, and each group shares the same rational function parameters. The operation is written as:

(19) $$\mathrm{GR}-\mathrm{KAN}\left(x\right)=\Phi \circ x=\left[\sum_{i=1}^{d_{\mathrm{in}}}w_{i,1}F_{\left\lfloor i/d_{g}\right\rfloor }\left(x_{i}\right),\ldots ,\sum_{i=1}^{d_{\mathrm{in}}}w_{i,d_{\mathrm{out}}}F_{\left\lfloor i/d_{g}\right\rfloor }\left(x_{i}\right)\right]$$

Alternatively, in matrix form:

(20) $$\mathrm{GR}-\mathrm{KAN}\left(x\right)=WF\left(x\right)=\left[\begin{array}{ccc} w_{1,1} & \ldots & w_{1,d_{\mathrm{in}}}\\ \vdots & \ddots & \vdots \\ w_{d_{\mathrm{out}},1} & \ldots & w_{d_{\mathrm{out}},d_{\mathrm{in}}} \end{array}\right]\cdot \left[\begin{array}{c} F_{\left\lfloor 1/d_{g}\right\rfloor }\left(x_{1}\right)\\ \vdots \\ F_{\left\lfloor d_{\mathrm{in}}/d_{g}\right\rfloor }\left(x_{d_{\mathrm{in}}}\right) \end{array}\right]$$

This can be implemented as:

(21) GR-KAN(x)=linear(group(x))

To ensure training stability, the parameters are initialized by estimating:

(22) $$\alpha =E\left[F{\left(x\right)}^{2}\right]\cdot \mathrm{Var}\left[x\right]\;\Rightarrow \;w∼N\left(0,\frac{\alpha }{d_{\mathrm{in}}}\right)$$

This ensures that Var[ϕ(x)]=Var[x], stabilizing the learning process.

### 4.4. Classification Layer Module

To achieve the final classification of motor imagery EEG signals, we design a classification module based on GR-KAN at the end of the model. This module is responsible for receiving the sequential features output by the attention encoder and mapping them to the category space. Since high-dimensional sequential features are inherently difficult to classify directly, and traditional MLPs with linear structures often exhibit limited expressive power when dealing with non-stationary and multi-scale EEG patterns—failing to capture discriminative structural information—we adopt the idea of GR-KAN and introduce the GroupKANLinear structure. This structure follows a “nonlinear activation first, then linear transformation” approach, utilizing learnable rational functions to enhance the model’s capacity to fit complex EEG patterns.

Specifically, we first perform a residual connection (element-wise addition) between the output features from LG-KAT, denoted as $X_{\mathrm{att}}\in R^{B\times L\times D}$, and the initial embedded features from MPE-CNN, denoted as $X_{\mathrm{embed}}\in R^{B\times L\times D}$, to form a fused representation:

(23) $$X_{\mathrm{fused}}=X_{\mathrm{embed}}+X_{\mathrm{att}}$$

This operation not only preserves the fundamental information from the initial features but also incorporates the contextual semantics modeled by the attention mechanism, resulting in a semantically richer input representation.

Next, we flatten the fused 3D tensor $X_{\mathrm{fused}}$ into a 1D vector:

(24) $$x=\mathrm{Flatten}\left(X_{\mathrm{fused}}\right)\in R^{B\times (L\cdot D)}$$

This vector aggregates contextual information across all time steps, serving as the final high-dimensional feature representation for each sample.

Subsequently, we proceed with the equations from Equations (18)–(22). This design not only improves the model’s expressive power but also reduces the parameter size from $O(d_{\mathrm{in}}\times d_{\mathrm{out}})$ to $O(\left(d_{\mathrm{in}}/g\right)\times d_{\mathrm{out}}+m+n)$, where *m* and *n* are the degrees of the numerator and denominator polynomials, respectively. Here, m and n are fixed at default values of 5 and 4.

During training, we use the cross-entropy loss function as the optimization objective:

(25) $$L=-\frac{1}{M}\sum_{i=1}^{M}\sum_{j=1}^{N}y_{ij}\log\left({\hat{y}}_{ij}\right)$$

where *M* denotes the number of MI-EEG trials, *N* is the number of classes, $y_{ij}$ is the ground-truth label for the *j*-th class of the *i*-th sample, and ${\hat{y}}_{ij}$ is the predicted probability for that class.

**Figure 4:**
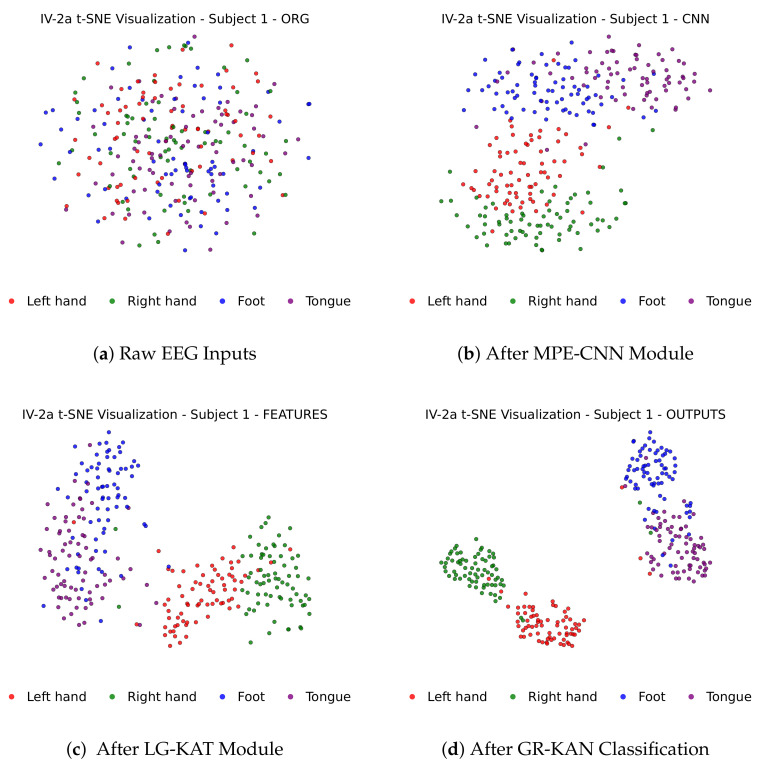
t-SNE visualization of feature evolution across the MCTGNet pipeline.

Figure 4 illustrates how MI-EEG features evolve through each stage of the MCTGNet architecture, showing progressively improved class separability from raw inputs to final classification outputs via t-SNE projections.

## 5. Performance Evaluation

### 5.1. Evaluation Setup

We implemented MCTGNet using Python 3.9 and PyTorch 2.1.2, running on Ubuntu 22.04 with an NVIDIA GeForce RTX 4090 GPU. We employed the Adam optimizer with a fixed learning rate of 0.001 throughout the training process. The optimizer parameters were set as $\beta_{1}=0.5$ and $\beta_{2}=0.999$, which were selected based on a grid search for optimal performance in similar tasks. We set the number of training epochs to 2000 and a batch size of 72. Cross-entropy loss was used as the objective function, and model parameters were updated via backpropagation.

A subject-dependent evaluation strategy was adopted, where the model is independently trained and tested for each subject, ensuring no data leakage between training and test sets. To evaluate performance, we reported both classification accuracy and standard deviation (Std), which together reflect the model’s effectiveness and robustness across subjects.

#### 5.1.1. Dataset

We conducted experiments on three publicly available motor imagery (MI) EEG datasets: **BCI Competition IV-2a** (IV-2a) [45], **BCI Competition IV-2b** (IV-2b) [46], and **OpenBMI** [47]. All datasets are widely used for benchmarking MI-EEG decoding models. IV-2a and IV-2b were both sampled at 250 Hz and preprocessed using a 0.5–100 Hz bandpass filter and a 50 Hz notch filter. For each trial, a four-second segment of motor-relevant EEG was extracted, resulting in a feature matrix of size C×1000, where *C* denotes the number of channels. IV-2a comprises 22-channel EEG recordings across two sessions for four-class classification (left hand, right hand, feet, and tongue), IV-2b contains 3-channel EEG recordings from five sessions for binary classification (left vs. right hand), while OpenBMI comprises 62-channel EEG recordings across two sessions for binary classification (left hand, right hand). The original 1000 Hz signals of OpenBMI were downsampled to 250 Hz, aligning with the sampling rate of IV-2a and IV-2b. In our experiments, earlier sessions were used for training and later sessions for testing. Key details of both datasets are summarized in Table 2.

#### 5.1.2. Data Augmentation

**Figure 5:**
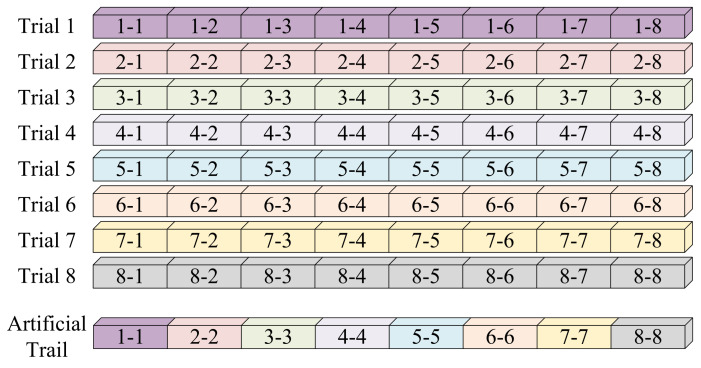
The principle of data augmentation.

In MI-EEG decoding tasks, the number of training samples for each subject is often limited, which poses a risk of overfitting and hinders the learning of robust representations. To solve this problem, MCTGNet adopts a class-consistent data augmentation strategy [48] based on time-series slicing and recombination (S&R). As illustrated in Figure 5, each EEG trial is first divided into eight equal-length segments (each 500 ms or 125 time points). Then, new synthetic trials are generated by randomly selecting segments from different trials of the same class and concatenating them in chronological order. This process increases sample diversity while preserving essential class characteristics.

### 5.2. Overall Performance

To comprehensively evaluate the effectiveness of the MCTGNet model, we conducted repeated experiments against several state-of-the-art methods, including EEGNet [19], Conformer [27], ATCNet [49], CTNet [50], and TMSA-Net [51]. All models were evaluated under consistent experimental settings, such as identical data preprocessing procedures, train–test splits, and data augmentation strategies.

**Performance on IV-2a Dataset:** As shown in Table 3, MCTGNet achieved the highest average classification accuracy of 88.93% on the IV-2a four-class dataset, outperforming the second-best model ATCNet (85.61%) by a margin of 3.32% and EEGNet (77.74%) by 11.19%. Notably, MCTGNet also achieved the lowest standard deviation (7.64), indicating superior consistency and robustness across subjects.

At the individual level, MCTGNet attained outstanding accuracies for subjects A03 (97.57%), A07 (96.53%), and A09 (92.71%), reflecting strong adaptability to typical neural response patterns. Even in subjects with low signal-to-noise ratios (e.g., A02 and A06), it still surpassed prior methods by a significant margin. These results confirm the strong classification capability of MCTGNet, which largely stems from its integrated multi-scale feature encoding and the advanced classifier design tailored for MI-EEG decoding.

**Performance on IV-2b Dataset: **Table 4 presents the results for the binary classification task on the IV-2b dataset. MCTGNet again achieved the highest average accuracy of 91.42%, outperforming CTNet (89.59%) and EEGNet (87.98%) by margins of 1.83% and 3.44%, respectively. Its standard deviation (7.91) was also the lowest, indicating reliable performance across subjects.

In particular, MCTGNet yielded nearly perfect accuracies for subjects B04, B05, and B08, demonstrating its ability to effectively model well-defined MI-EEG patterns. For more challenging cases, such as B02, MCTGNet still achieved 74.29%, surpassing EEGNet (69.29%) and ATCNet (70.35%). This also confirms the model’s robustness in capturing discriminative features under subject variability and noisy conditions.

**Performance on OpenBMI Dataset:** To further evaluate the generalization capability of MCTGNet, we conducted experiments on the OpenBMI dataset—a binary classification benchmark comprising 54 subjects with diverse data characteristics and experimental setups. This dataset poses a stringent challenge for cross-subject generalization. As shown in Table 5, MCTGNet achieved an average accuracy of 80.07%, outperforming CTNet (77.96%) by 2.11%, and significantly surpassing EEGNet (74.22%) and ATCNet (75.23%). Notably, MCTGNet also achieved the lowest standard deviation (13.29%), indicating strong robustness under heterogeneous conditions.

**Figure 6:**
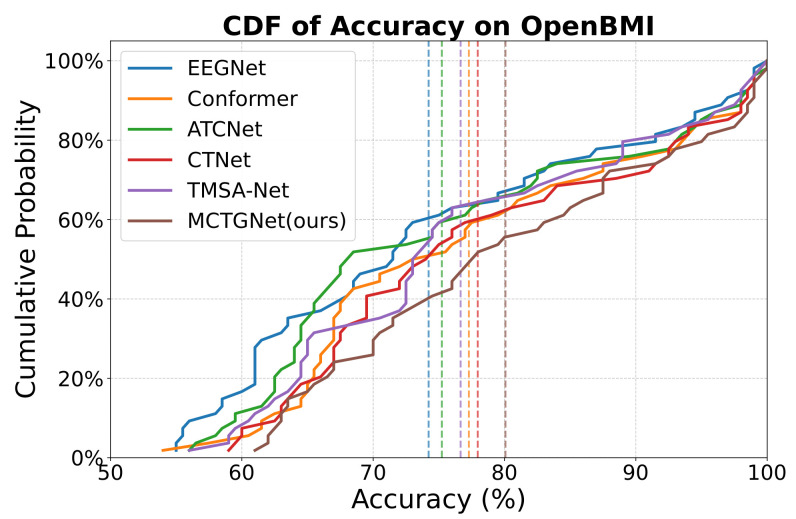
Subject-wise accuracy distribution (CDF) on OpenBMI Dataset.

The cumulative distribution function (CDF) is shown in Figure 6 to further characterize MCTGNet’s performance distribution across subjects. It reveals that MCTGNet maintains a clear advantage at key percentiles: at the 50th percentile, it outperforms the second-best method by approximately 3%; at the 80th percentile, the margin increases to over 4%; and at the 90th percentile, MCTGNet leads by nearly 5%. These results demonstrate that MCTGNet not only delivers high average performance but also generalizes consistently across a broad range of subjects, particularly in the high-accuracy regime.

**Figure 7:**
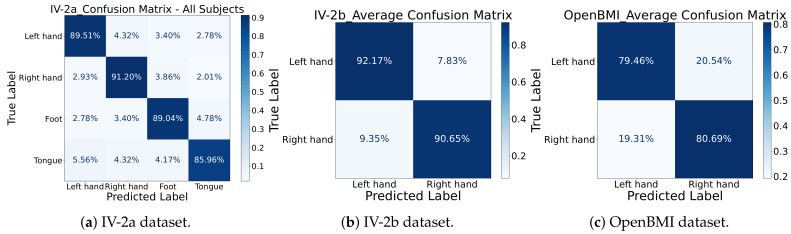
Average confusion matrix of MCTGNet on IV-2a, IV-2b, and OpenBMI datasets.

**Confusion Matrix Analysis:** To further examine the discriminative performance of MCTGNet, we visualized the average confusion matrices for all datasets in Figure 7. On IV-2a, the classification accuracies for the four classes—left hand, right hand, feet, and tongue—were 89.51%, 91.20%, 89.04%, and 85.96%, respectively. The most frequent confusion occurred between left and right hand imagery, a known challenge in MI-EEG decoding. On IV-2b, left-hand imagery was correctly identified with 92.17% accuracy and right-hand imagery with 90.65%. For the OpenBMI dataset, left-hand imagery achieves a correct identification rate of 79.46%, with 20.54% misclassified as right-hand; right-hand imagery has an 80.69% correct classification rate, and 19.31% are misidentified as left-hand. The low misclassification rates further affirm MCTGNet’s capacity to differentiate between closely related EEG patterns.

### 5.3. Ablation Experiments

To assess the effectiveness and necessity of each core module within MCTGNet, we conducted systematic ablation experiments to quantitatively evaluate the contribution of individual components to the model’s overall performance. We tested five ablation settings. The performance metrics are summarized in Table 6 and Table 7.

- Removing the time-series slicing and recombination data augmentation strategy (No Augmentation),
- Replacing the multi-scale convolutional structure with a single-scale version (No Multi-Scale CNN),
- Excluding the LG-KAT module (No LG-KAT),
- Replacing the GR-KAN with conventional MLPs (No GR-KAN),
- Simultaneously removing both the multi-scale CNN and LG-KAT (No Multi-Scale CNN & LG-KAT).

From Table 6, we observe that the full MCTGNet model achieves the best performance on IV-2a with 88.93% average accuracy and the lowest standard deviation of 7.64. Removing the data augmentation module leads to a performance drop to 84.72%, particularly for subject A02, which drops from 79.51% to 73.61%. This indicates the effectiveness of the S&R strategy in improving generalization, especially for noisy data.

Removing the GR-KAN module reduces accuracy to 86.50%, and for subject A06, the drop is significant (from 75.35% to 71.88%), demonstrating GR-KAN’s superior ability in modeling nonlinear features. Similarly, removing the multi-scale CNN module leads to a larger decline (82.99% overall, with A02 and A06 dropping to 70.14% and 64.58%), highlighting the importance of multi-scale temporal receptive fields in EEG feature extraction.

As shown in Table 7, the full model also yields the best results on IV-2b. When comparing with ablated versions, all removed components cause a performance drop. Notably, GR-KAN improves separability even in a simpler binary classification setting, as B06’s accuracy improves from 89.38% (without GR-KAN) to 91.25%.

**Figure 8:**
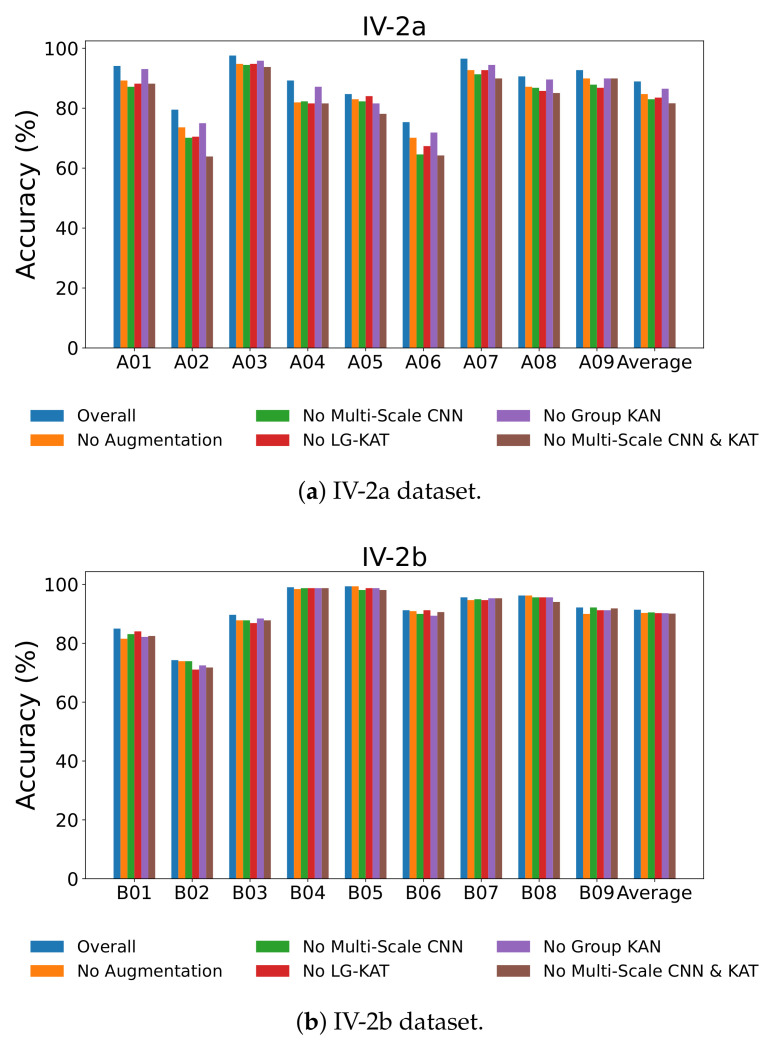
Performance comparison of different architecture configurations in ablation experiments.

Figure 8 visually confirms these results across all subjects, showing consistent performance drops when individual modules are removed. The most drastic degradation occurs when both the multi-scale CNN and LG-KAT are removed, emphasizing their complementary roles in hierarchical temporal modeling.

**Figure 9:**
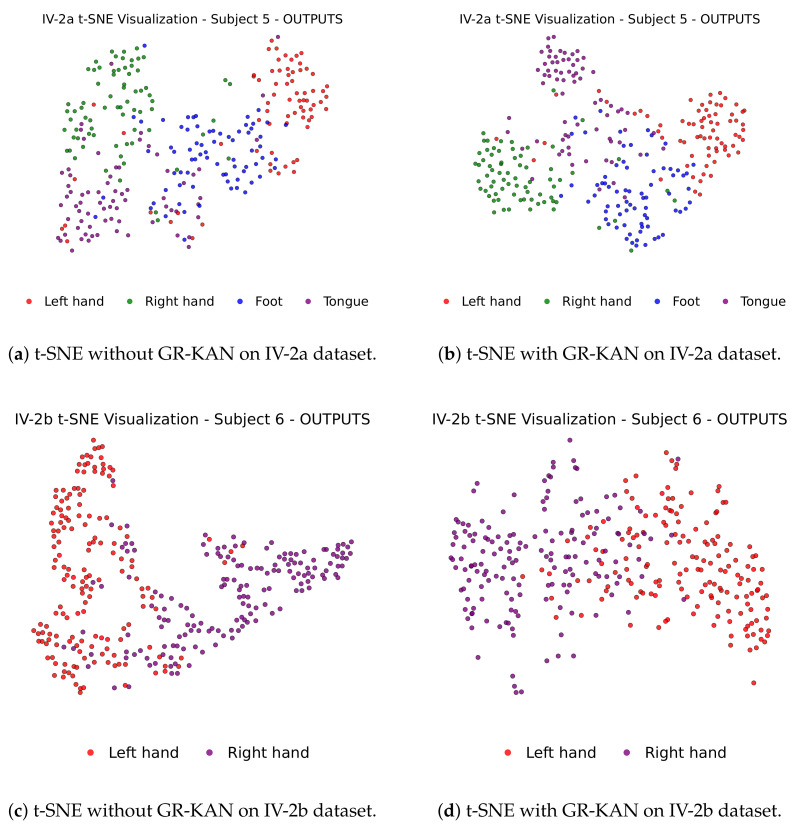
t-SNE visualization of features before and after introducing GR-KAN. Top: Subject 5 (IV-2a); Bottom: Subject 6 (IV-2b). GR-KAN results in more compact and discriminative clusters.

To further investigate the impact of GR-KAN on the learned representation quality, we apply t-SNE to visualize the distribution of features before classification. Figure 9 displays the 2D feature projections of selected subjects from IV-2a and IV-2b, with and without GR-KAN.

In the absence of GR-KAN (Figure 9a,c), different class clusters show significant overlap, particularly in complex subjects. In contrast, with GR-KAN integrated (Figure 9b,d), features become more compact and separable, clearly delineating category boundaries. This confirms GR-KAN’s capacity to enhance semantic separability and structural clustering in the latent feature space.

These ablation results collectively demonstrate that each module in MCTGNet—data augmentation, multi-scale convolution, LG-KAT, and GR-KAN—plays a crucial and irreplaceable role in boosting decoding performance and generalization in MI-EEG tasks.

### 5.4. Classifier Performance Comparison

To evaluate the effectiveness of GR-KAN as the classification head and the nonlinear layers in MCTGNet, we replace it with several commonly used alternatives, including MLPs, polynomial kernel-based SVMs, and conventional KANs. Key metrics such as classification accuracy, training time, and test loading time are analyzed.

As shown in Table 8, GR-KAN achieves the highest classification accuracy across both datasets, reaching 88.93% on IV-2a and 91.42% on IV-2b—representing relative gains of 2.43% and 1.12% over the next best-performing method. Meanwhile, GR-KAN consistently delivers superior performance in both IV-2a and IV-2b datasets, demonstrating its ability to effectively model complex EEG patterns while maintaining lower test loading times and more efficient training. This makes GR-KAN a promising solution for real-time BCI applications.

### 5.5. Impact Factors

This section investigates the influence of critical hyperparameters on the model’s classification performance in a subject-dependent setting. Specifically, we explore the impact of (1) different combinations of multi-scale convolution kernel sizes, (2) the number of LG-KAT layers, and (3) the group number in the GR-KAN module.

#### 5.5.1. Effect of Convolution Kernel Size Combinations

**Figure 10:**
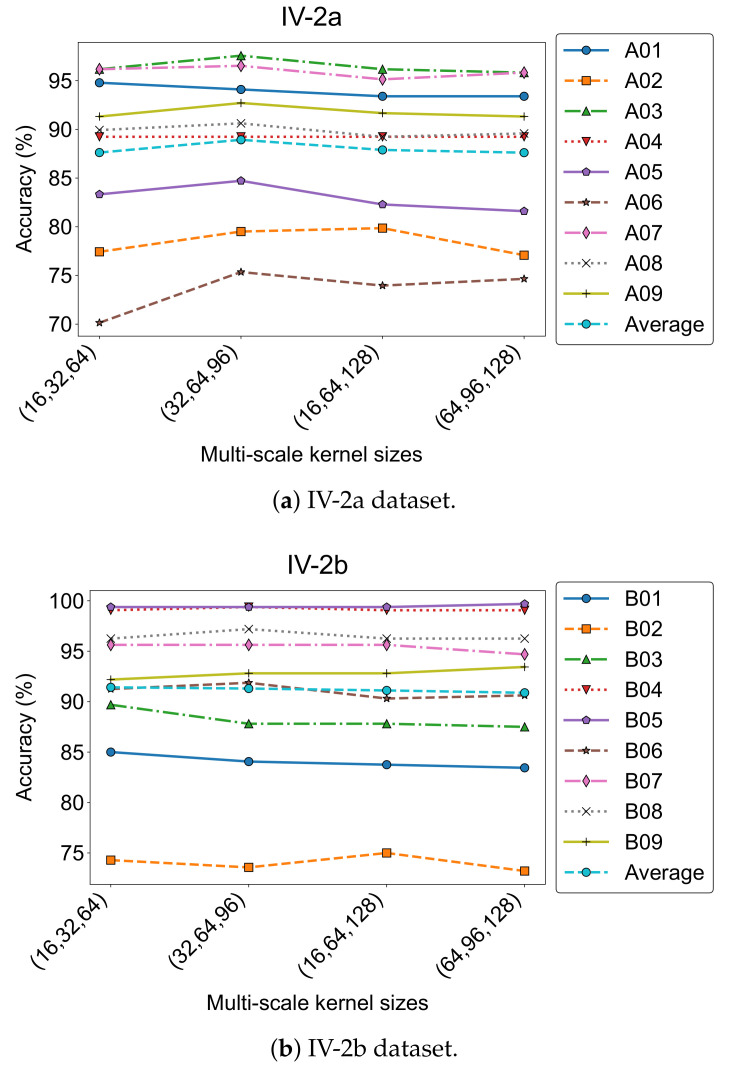
Classification accuracy of MCTGNet with different kernel size combinations.

To evaluate how different temporal receptive field settings affect model performance, we tested four kernel size combinations in the multi-scale convolution module: (16, 32, 64), (32, 64, 96), (16, 64, 128), and (64, 96, 128). The results are shown in Figure 10, where accuracy trends across subjects are plotted for both datasets.

On the IV-2a dataset, the combination (32, 64, 96) achieved the highest average accuracy (88.93%) and the lowest standard deviation (7.64). For IV-2b, the best performance (91.42%) was obtained with (16, 32, 64). These results were adopted as the default settings in our final model.

Despite the strong average performance, individual differences remained notable. For instance, Subject A01 performed best with (16, 32, 64) (94.79%), while A02 preferred (16, 64, 128) (79.86%). Similarly, B01 and B03 in IV-2b achieved top accuracies using the (16, 32, 64) configuration. This highlights the variability in optimal kernel scales, likely due to inter-subject differences in temporal and spectral characteristics of MI-EEG signals.

#### 5.5.2. Effect of the Number of LG-KAT Layers

**Figure 11:**
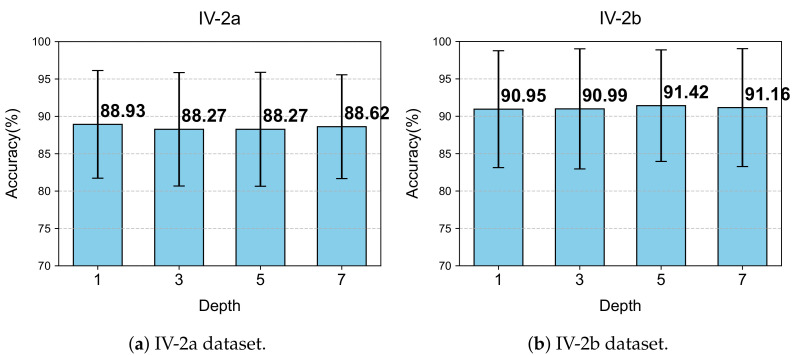
Performance of MCTGNet with different numbers of LG-KAT layers.

We further evaluated how varying the number of LG-KAT layers influences classification accuracy. Four configurations were tested: 1, 3, 5, and 7 layers. The experimental results are illustrated in Figure 11.

For IV-2a, the best accuracy (88.93%) was obtained with only one LG-KAT layer, along with the lowest standard deviation (7.64). Increasing the number of layers slightly degraded the performance, with only marginal accuracy differences observed. Additionally, more layers led to longer training times.

In contrast, IV-2b benefited slightly from deeper attention modeling: five layers yielded the highest accuracy (91.42%), outperforming the single-layer configuration by 0.47%. Given the lower complexity of binary classification, this trade-off was considered acceptable. Hence, we adopted one layer for IV-2a and five layers for IV-2b in the final model, balancing efficiency and effectiveness across task types.

#### 5.5.3. Effect of Group Number in GR-KAN

**Figure 12:**
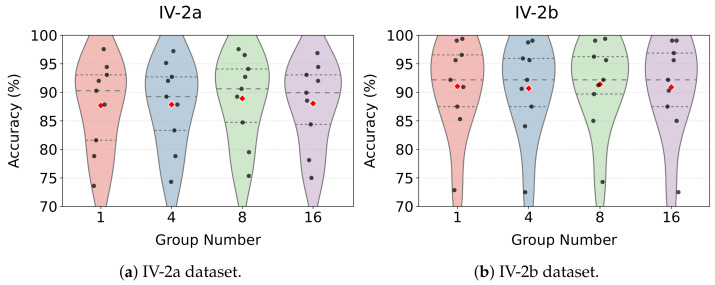
Classification performance of MCTGNet under different group numbers in GR-KAN. Red dots indicate the mean accuracy across subjects.

The group number in GR-KAN determines how the input channels are partitioned for rational function learning and plays a crucial role in balancing expressiveness and computational cost. We examined group values of 1, 4, 8, and 16 on both datasets. The subject-wise and average accuracies are shown in Figure 12.

For IV-2a, the average accuracy increased from 87.69% (Group = 1) to 88.93% (Group = 8), with a concurrent drop in standard deviation from 8.00 to 7.64. Notably, Subjects A01, A03, and A07 achieved top accuracies at Group = 8. Similar trends were observed in IV-2b, where Group = 8 led to the best average accuracy of 91.42%, outperforming both Group = 1 and Group = 16.

These results suggest that Group = 8 achieves the best trade-off between parameter sharing and feature representation. In contrast, setting the group number too high (e.g., 16) can overly fragment the input features, thereby impairing the model’s ability to learn coherent patterns and degrading performance.

## 6. Discussion

The proposed MCTGNet model significantly improves classification accuracy and cross-session generalization in MI-EEG decoding by integrating multi-scale convolutional modules, hybrid attention mechanisms, and the GR-KAN classifier. Although the model achieves strong performance across multiple public datasets, its robustness still has room for improvement—for instance, whether it can demonstrate consistent performance gains in cross-subject scenarios remains to be validated.

First, while the multi-scale feature extraction module effectively captures neural rhythms across different frequency bands, the frequency components induced by MI vary considerably among individuals. As a result, fixed convolutional kernel combinations may fail to fully capture critical subject-specific neural variations. Second, we confirm that the integration of GR-KAN enhances nonlinear modeling capabilities. Although its computational efficiency significantly outperforms conventional KANs, its structural complexity still exceeds that of traditional MLPs. Furthermore, during the training phase, computational overhead may vary depending on GR-KAN’s parameter configurations, suggesting potential avenues for optimizing its efficiency.

Moreover, dataset bias remains a notable issue. Existing EEG datasets predominantly focus on specific population groups, and EEG signals exhibit substantial variability across different time points, physiological states, and psychological conditions. These factors may undermine the model’s temporal stability and generalization. To address this, future work will involve constructing a more diverse, self-collected MI-EEG dataset to improve adaptability across demographic variations and session intervals.

In addition, integrating MCTGNet with other physiological modalities, such as eye movement and electromyography (EMG), is expected to extend its decoding capacity under multimodal input settings, thereby further enhancing robustness and discriminative power. To meet the requirements of large-scale real-time BCI systems, future efforts will also focus on optimizing model architecture and inference strategies to ensure low-latency performance. These efforts collectively pave the way for applying MCTGNet to large-scale, real-time brain–computer interface systems.

## 7. Conclusions

This paper presents MCTGNet, a novel end-to-end deep neural network for MI-EEG decoding. The proposed architecture combines a multi-scale convolutional module for extracting temporal features across diverse frequency bands, a local-global attention encoder based on the Kolmogorov–Arnold Transformer for modeling both local and long-range dependencies, and a GR-KAN-based classification layer that enhances nonlinearity and representational capacity. Evaluations on the BCI Competition IV-2a and IV-2b datasets show that MCTGNet achieves average accuracies of 88.93% and 91.42%, respectively, outperforming state-of-the-art methods by 3.32% and 1.83%.

## Figures and Tables

**Table 1:** Hyperparameter settings in the multi-scale feature extraction module.

Parameter	Value	Description
$F_{1}$	8	Number of filters in temporal convolution branch
*D*	2	Depth multiplier in channel depth-wise convolution
$K_{1}=\left\{K_{S},K_{M},K_{L}\right\}$	{32, 64, 96}	Multi-scale kernel sizes (short/medium/long)
$P_{1}$	8	Pooling size for first temporal downsampling
$K_{2}$	16	Kernel size for spatial dimension convolution
$P_{2}$	8	Pooling size for second temporal downsampling

**Table 2:** The information about the datasets.

	IV-2a	IV-2b	OpenBMI
Number of Subjects	9	9	54
Number of Sessions	2	5	2
Number of Channels	22	3	62
Length of Time points	1000	1000	4000
Frequency of Samples	250 Hz	250 Hz	1000 Hz
Number of Classes	4	2	2
Size of Train Datas	288 × 22 × 1000	400 × 3 × 1000	100 × 62 × 1000
Size of Test Datas	288 × 22 × 1000	320 × 3 × 1000	100 × 62 × 1000

**Table 3:** Comparison of the proposed method with other state-of-the-art methods on IV-2a dataset.

	A01	A02	A03	A04	A05	A06	A07	A08	A09	Average	Std
EEGNet	86.81	67.36	91.32	66.67	73.96	68.06	82.64	78.82	84.03	77.74	9.15
Conformer	82.99	54.86	94.79	73.96	76.38	62.50	85.07	88.54	81.94	77.89	12.64
ATCNet	87.85	71.18	95.83	82.99	82.64	**76.39**	95.83	90.62	87.15	85.61	8.29
CTNet	91.67	73.96	96.88	85.07	81.60	65.97	93.40	88.19	86.46	84.80	9.77
TMSA-Net	84.38	64.58	95.49	83.68	79.51	66.67	92.71	88.54	83.68	82.14	10.58
**MCTGNet (ours)**	**94.10**	**79.51**	**97.57**	**89.24**	**84.72**	75.35	**96.53**	**90.62**	**92.71**	**88.93**	**7.64**

Note: **Bold** denotes the best performance in this table.

**Table 4:** Comparison of the proposed method with other state-of-the-art methods on IV-2b dataset.

	B01	B02	B03	B04	B05	B06	B07	B08	B09	Average	Std
EEGNet	78.75	69.29	87.19	98.75	93.43	89.38	93.75	94.06	87.19	87.98	9.04
Conformer	80.93	**74.64**	85.31	98.44	98.75	87.50	92.81	93.13	88.75	88.92	7.93
ATCNet	80.31	70.35	86.25	98.13	97.81	90.63	95.31	91.88	89.38	88.89	8.96
CTNet	79.37	70.71	86.88	98.75	98.13	90.31	95.00	95.62	91.56	89.59	9.32
TMSA-Net	82.50	71.07	87.50	98.13	98.44	89.37	93.13	95.63	87.50	89.25	8.67
**MCTGNet (ours)**	**85.00**	74.29	**89.69**	**99.06**	**99.38**	**91.25**	**95.63**	**96.25**	**92.19**	**91.42**	**7.91**

Note: **Bold** denotes the best performance in this table.

**Table 5:** Comparison of the proposed method with other state-of-the-art methods on OpenBMI dataset.

Method	Average	Std
EEGNet	74.22	14.59
Conformer	77.29	13.75
ATCNet	75.23	14.49
CTNet	77.96	13.55
TMSA-Net	76.66	13.57
**MCTGNet (ours)**	**80.07**	**13.29**

Note: **Bold** denotes the best performance in this table.

**Table 6:** Performance of ablation study on IV-2a dataset.

Augmentation	Multi-Scale CNN	LG-KAT	GR-KAN	Accuracy	Std
✓	×	×	✓	81.64	11.01
×	✓	✓	✓	84.72	8.41
✓	×	✓	✓	82.99	9.75
✓	✓	×	✓	83.53	9.25
✓	✓	✓	×	86.50	8.55
✓	✓	✓	✓	**88.93**	**7.64**

Note: ✓ means the corresponding module is adopted; × means the corresponding module is not adopted; **Bold** denotes the best performance in this table.

**Table 7:** Performance of ablation study on IV-2b dataset.

Augmentation	Multi-Scale CNN	LG-KAT	GR-KAN	Accuracy	Std
✓	×	×	✓	90.09	8.54
×	✓	✓	✓	90.33	8.32
✓	×	✓	✓	90.51	8.00
✓	✓	×	✓	90.26	8.75
✓	✓	✓	×	90.33	8.32
✓	✓	✓	✓	**91.42**	**7.91**

Note: ✓ means the corresponding module is adopted; × means the corresponding module is not adopted; **Bold** denotes the best performance in this table.

**Table 8:** Performance comparison of different classifiers.

Performance Indicators	MLPs		SVMs		KANs		GR-KAN	
	**IV-2a**	**IV-2b**	**IV-2a**	**IV-2b**	**IV-2a**	**IV-2b**	**IV-2a**	**IV-2b**
Model Total Parameter Size (MB)	**9.47**	**2.91**	9.63	**2.91**	9.64	2.94	9.68	3.16
Training Time per Epoch (epoch/s)	0.613	**0.476**	0.686	0.525	0.806	0.889	**0.601**	0.617
Model Test Loading Time (ms)	53.5	**126.5**	51.1	126.7	435	1001.4	**35.8**	191.2
Average Classification Accuracy (%)	86.50	90.24	84.65	90.30	84.49	90.24	**88.93**	**91.42**

Note: **Bold** denotes the best performance in this table.

## Data Availability

The datasets used in this study are publicly available. The IV-2a dataset can be accessed at https://www.bbci.de/competition/download/competition_iv/BCICIV_2a_gdf.zip (accessed on 9 October 2024), the IV-2b dataset is available at https://www.bbci.de/competition/download/competition_iv/BCICIV_2b_gdf.zip (accessed on 9 October 2024), and the OpenBMI dataset is available at https://gigadb.org/dataset/view/id/100542/Files_page/1 (accessed on 15 June 2025). No new data were generated in this study.
